# Respiratory event duration reveals novel phenotypes beyond the apnea–hypopnea index in obstructive sleep apnea

**DOI:** 10.3389/fmed.2026.1960647

**Published:** 2026-09-29

**Authors:** Mo Chen, Jing Yang, Yang Yang, Zijing Wang, Youqing Lai, Huijia Lei

**Affiliations:** Department of Otorhinolaryngology, Beijing Jishuitan Hospital Affiliated to Capital Medical University, Beijing, China

**Keywords:** apnea- hypopnea index (AHI), cluster analysis, obstructive sleep apnea, phenotype, polysomnography, respiratory event duration

## Abstract

**Background:**

Obstructive sleep apnea (OSA) is a heterogeneous disorder characterized by recurrent upper airway collapse during sleep. Although the apnea–hypopnea index (AHI) remains the cornerstone of OSA diagnosis and severity assessment, it primarily reflects the frequency of respiratory events and may not adequately capture differences in respiratory event duration or the associated physiological burden. We aimed to identify novel OSA phenotypes by incorporating respiratory event duration into polysomnography-based cluster analysis.

**Methods:**

We retrospectively analyzed consecutive adult patients with OSA who underwent overnight polysomnography. Demographic, anthropometric, and polysomnographic characteristics were collected. Age, body mass index (BMI), neck circumference, AHI, lowest oxygen saturation (LowSpO₂), longest apnea duration (LAP), and longest hypopnea duration (LHP) were included in the clustering analysis. Hierarchical clustering using Ward’s method was first performed to determine the optimal number of clusters, followed by K-means clustering to identify distinct OSA phenotypes.

**Results:**

Among 999 consecutive patients, 959 with complete clustering variables were included in the final analysis. Hierarchical clustering followed by K-means clustering identified four distinct OSA phenotypes. Cluster 1 represented a mild phenotype characterized by the lowest AHI, the shortest LAP, and preserved nocturnal oxygenation. Cluster 2 represented an obesity-associated severe phenotype with the highest BMI, the largest neck circumference, the highest AHI, and the greatest degree of nocturnal oxygen desaturation. Cluster 3 was characterized by markedly prolonged apnea duration despite only moderate AHI, whereas Cluster 4 exhibited the longest apnea and hypopnea durations accompanied by pronounced nocturnal hypoxemia. Significant differences were observed among the four phenotypes in demographic characteristics, obesity-related measures, disease severity, nocturnal oxygenation, and respiratory event duration (all *P* < 0.001). Importantly, respiratory event duration provided additional temporal characterization across phenotypes beyond conventional frequency-based severity assessment.

**Conclusions:**

Respiratory event duration–enhanced clustering identified four exploratory OSA phenotypes with distinct temporal characteristics. These findings suggest that respiratory event duration may provide complementary information to conventional AHI-based severity assessment, while its clinical utility requires further validation.

## Introduction

Obstructive sleep apnea (OSA) is one of the most prevalent sleep-related breathing disorders and is characterized by recurrent upper airway collapse during sleep, leading to intermittent hypoxia, sleep fragmentation, and recurrent arousals (1, 2). OSA is associated with an increased risk of hypertension, cardiovascular disease, stroke, metabolic dysfunction, neurocognitive impairment, and impaired quality of life (3–7). Long-term cohort studies have further highlighted the substantial health burden associated with untreated OSA (8). With an estimated one billion adults affected worldwide, OSA has become a major global public health challenge. Overnight polysomnography (PSG) remains the diagnostic gold standard, with disease severity conventionally quantified using the apnea–hypopnea index (AHI), defined as the average number of apnea and hypopnea events per hour of sleep (9).

Despite its widespread use as the cornerstone of OSA diagnosis and severity classification, accumulating evidence indicates that the AHI does not fully capture the complexity of the disease (10). By design, the AHI reflects only the frequency of respiratory events and assigns equal importance to all apneas and hypopneas regardless of their duration or physiological consequences (11, 12). As a result, patients with comparable AHI values may experience markedly different degrees of oxygen desaturation, sleep fragmentation, and cardiovascular stress (13, 14). Accordingly, increasing attention has been directed toward complementary physiological metrics, including hypoxic burden, arousal characteristics, and respiratory event morphology, to improve disease characterization and prediction of clinical outcomes (15–17).

The marked clinical heterogeneity of OSA has stimulated growing interest in phenotype-based classification (18, 19). Previous clustering studies have shown that patients with similar AHI values may exhibit distinct clinical characteristics, symptom profiles, obesity patterns, and cardiovascular risks (20, 21). Collectively, these studies indicate that conventional severity classification alone is insufficient to capture the biological and clinical diversity of OSA. To date, most clustering studies have focused primarily on demographic characteristics, anthropometric parameters, symptoms, and conventional polysomnographic indices, whereas respiratory event duration has rarely been incorporated into phenotype identification (20–22)..

Respiratory event duration has emerged as a potentially informative yet underexplored physiological characteristic of OSA (23). Whereas the AHI quantifies the frequency of respiratory events, the duration of individual apnea and hypopnea episodes may more directly determine cumulative hypoxic exposure and the associated physiological stress during sleep. To our knowledge, few studies have incorporated the longest apnea duration (LAP) and longest hypopnea duration (LHP) into phenotype classification. Against this background, we sought to determine whether integrating respiratory event duration with conventional demographic and polysomnographic variables could identify clinically meaningful OSA phenotypes using hierarchical and K-means cluster analyses. We further characterized the clinical and polysomnographic features of the identified phenotypes and explored the potential contribution of respiratory event duration to OSA phenotyping.

## Methods

### Study design and population

This retrospective observational study was conducted at the Department of Otorhinolaryngology, Beijing Jishuitan Hospital. The study was designed to identify polysomnography-based phenotypes of adult obstructive sleep apnea by incorporating respiratory event duration into cluster analysis.

The study protocol was approved by the Ethics Committee of Beijing Jishuitan Hospital (No. 2023-364-00), and the requirement for written informed consent was waived owing to the retrospective nature of the study.

Consecutive adult patients who underwent overnight diagnostic polysomnography between January 2020 and December 2023 at Beijing Jishuitan Hospital were retrospectively screened. Adult patients diagnosed with OSA according to the AASM criteria were eligible for inclusion. Patients with missing values in any clustering variable were excluded from the final analysis.

### Polysomnography

Overnight attended diagnostic polysomnography was performed using a CompuMedics system (CompuMedics, Australia) in the sleep laboratory of the Department of Otorhinolaryngology at Beijing Jishuitan Hospital. The recordings included electroencephalography, electrooculography, submental electromyography, airflow, thoracoabdominal respiratory effort, electrocardiography, and pulse oximetry. Sleep stages and respiratory events were manually scored by experienced sleep physicians in accordance with the American Academy of Sleep Medicine (AASM) Manual for the Scoring of Sleep and Associated Events (9). Obstructive apnea was defined as a ≥90% reduction in airflow lasting at least 10 s with persistent respiratory effort. Hypopnea was defined as a ≥30% reduction in airflow lasting at least 10 s accompanied by ≥4% oxygen desaturation. The apnea–hypopnea index (AHI) was calculated as the total number of apneas and hypopneas divided by the total sleep time (hours).

### Selection of clustering variables

Variables included in the cluster analysis were selected *a priori* based on their established clinical relevance and their ability to capture complementary dimensions of OSA. Age, body mass index (BMI), and neck circumference were selected to represent demographic characteristics and obesity-related anatomical features. The apnea–hypopnea index (AHI) and the lowest oxygen saturation (LowSpO₂) were selected to characterize disease severity and nocturnal hypoxemia. To complement conventional frequency-based measures, respiratory event duration was incorporated using the longest apnea duration (LAP) and the longest hypopnea duration (LHP). Whereas the AHI quantifies the frequency of respiratory events, LAP and LHP characterize their temporal dimension and may therefore provide complementary information regarding physiological burden and phenotypic heterogeneity.

### Cluster analysis

Continuous variables were standardized using Z-score transformation before clustering to eliminate differences in measurement scales. Hierarchical cluster analysis was first performed using Ward’s linkage method with squared Euclidean distance. The dendrogram and agglomeration coefficients were examined to identify a clinically interpretable candidate solution. Because the original analysis did not systematically compare multiple candidate cluster numbers or perform formal resampling-based stability analyses, the four-cluster solution was treated as an exploratory solution rather than a uniquely optimal solution. Subsequently, K-means clustering was conducted with four clusters to classify patients into distinct phenotypes. The original SPSS specification used a maximum of 10 iterations, a convergence criterion of 0, and the NOUPDATE option. The four-cluster solution was retained as an exploratory phenotype classification based on the hierarchical clustering structure, cluster size, and clinical interpretability. Formal resampling-based cluster stability and external validation were not performed in the original analysis.

### Statistical analysis

Continuous variables are presented as mean ± standard deviation (SD), and categorical variables are presented as frequencies and percentages. Comparisons among clusters were performed using one-way analysis of variance (ANOVA) for the continuous clustering variables and Pearson’s chi-square test for categorical variables when applicable. When the overall ANOVA was statistically significant, pairwise comparisons were performed using the Bonferroni correction. All statistical analyses were conducted using IBM SPSS Statistics for Windows, version 22.0 (IBM Corp., Armonk, NY, USA).

A two-sided *P* value < 0.05 was considered statistically significant.

### Missing data

Patients with missing values in any clustering variable were excluded from the cluster analysis using listwise deletion. Among the 999 screened patients, 40 were excluded: 34 had missing LAP, 7 had missing LHP, and 1 each had missing BMI, neck circumference, or lowest SpO₂; some patients had more than one missing variable. Multiple imputation was not performed because the primary clustering analysis was based on complete cases and the missingness was concentrated in variables required for phenotype construction. The potential for selection bias from complete-case analysis is acknowledged as a limitation.

## Results

### Study population

A total of 999 consecutive adult patients who underwent overnight diagnostic polysomnography between January 2020 and December 2023 were retrospectively screened. After excluding 40 patients with missing values in one or more clustering variables, 959 patients were included in the final cluster analysis (Figure 1). Among the 959 patients included in clustering, 773 were male, 173 were female, and sex was missing for 13 patients.

**Figure 1:**
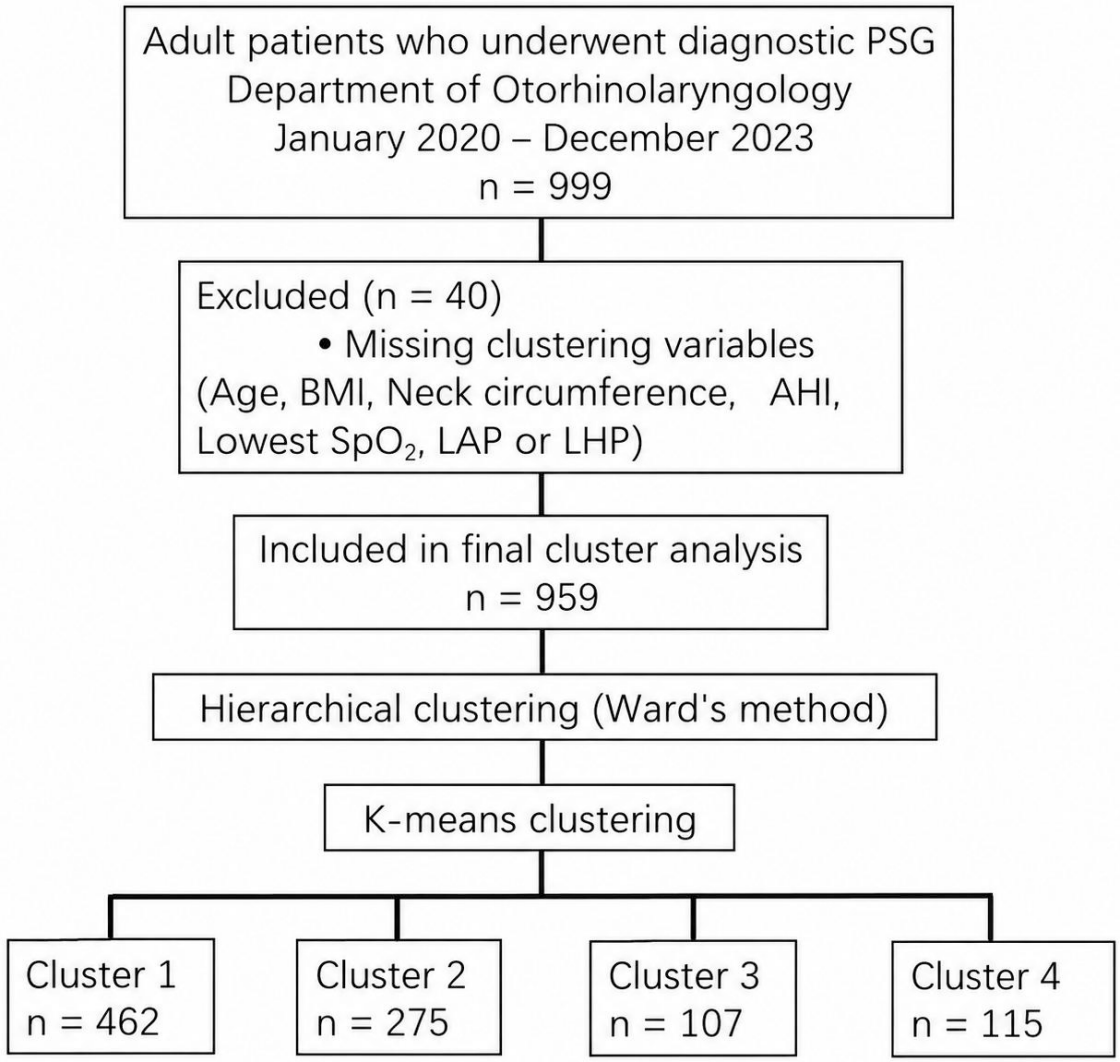
Flowchart of patient selection and clustering procedure. A total of 999 consecutive adult patients who underwent overnight diagnostic polysomnography between January 2020 and December 2023 were retrospectively screened. After excluding patients with missing clustering variables, 959 patients were included in the final analysis and classified into four phenotypes using hierarchical clustering followed by K-means clustering.

Hierarchical clustering followed by K-means clustering yielded four exploratory phenotypes. Cluster 1 comprised 462 patients (48.2%), Cluster 2 comprised 275 (28.7%), Cluster 3 comprised 107 (11.2%), and Cluster 4 comprised 115 (12.0%).

### Identification of the exploratory four-cluster solution

Hierarchical clustering using Ward’s linkage method supported the selection of a four-cluster exploratory solution based on the dendrogram structure, agglomeration coefficients, and clinical interpretability (Figure 2). Because multiple candidate cluster numbers were not systematically compared in the original analysis, four clusters are not described as a uniquely optimal solution.

**Figure 2:**
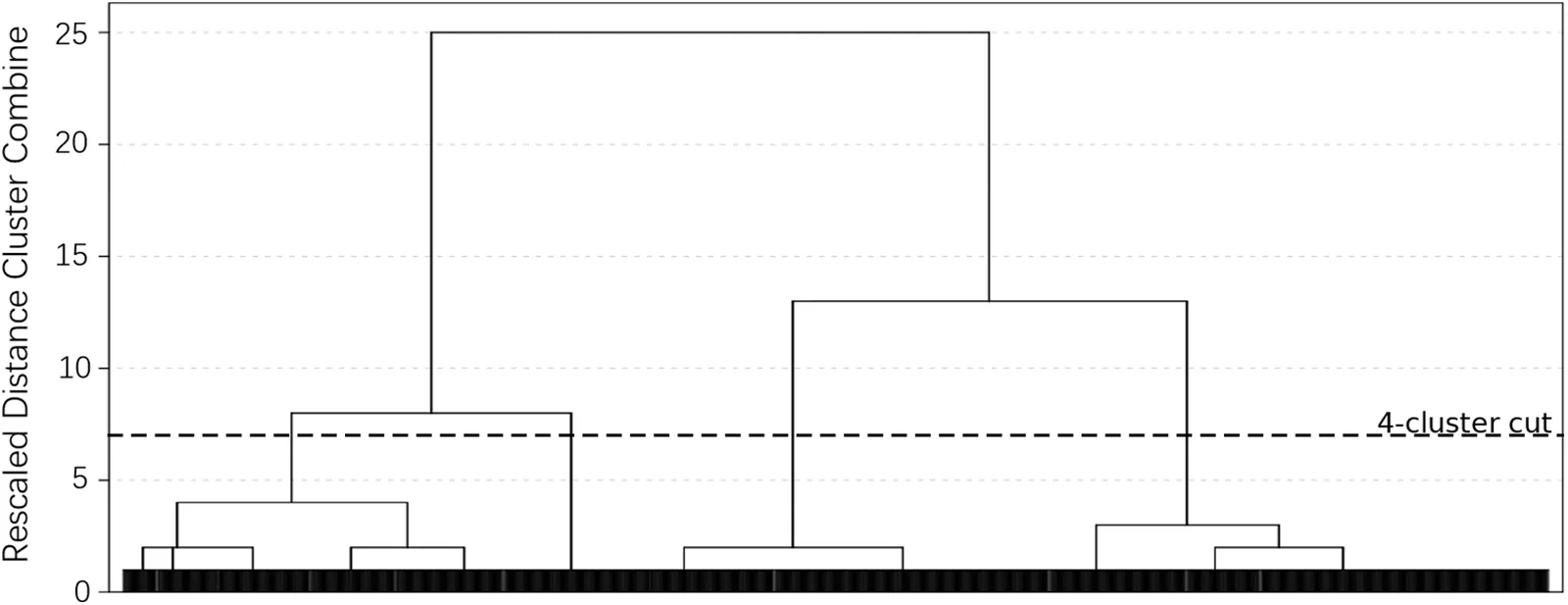
Dendrogram generated using ward’s hierarchical clustering with squared Euclidean distance. The four-cluster exploratory solution was selected based on the dendrogram structure, agglomeration coefficients, and clinical interpretability.

### Clinical characteristics of the four OSA phenotypes

K-means clustering identified four distinct phenotypes among the 959 adult patients with obstructive sleep apnea. Their demographic and polysomnographic characteristics are summarized in Table 1.

**Table 1:** Clinical and polysomnographic characteristics of the four obstructive sleep apnea phenotypes identified by cluster analysis.

Variable	Cluster 1 (*n* = 462)	Cluster 2 (*n* = 275)	Cluster 3 (*n* = 107)	Cluster 4 (*n* = 115)	*P* value
Demographic characteristics					
Age (years)	36.0 ± 11.4	40.8 ± 11.4	39.3 ± 12.0	41.5 ± 12.1	<0.001
BMI (kg/m²)	26.1 ± 4.6	29.0 ± 4.5	26.3 ± 3.9	27.0 ± 3.8	<0.001
Neck circumference (cm)	39.7 ± 5.8	42.7 ± 4.5	40.1 ± 5.7	40.5 ± 5.3	<0.001
Polysomnographic characteristics					
AHI (events/h)	7.1 ± 7.5	52.7 ± 23.0	18.9 ± 16.6	25.6 ± 18.6	<0.001
Hypopnea index (events/h)	4.9 ± 5.0	19.2 ± 10.9	7.4 ± 6.7	11.6 ± 9.0	<0.001
Lowest SpO₂ (%)	85.9 ± 6.3	72.1 ± 11.6	80.0 ± 10.7	75.1 ± 12.9	<0.001
Respiratory event duration					
LAP (s)	28.6 ± 12.0	64.8 ± 16.5	84.9 ± 21.0	124.9 ± 19.9	<0.001
LHP (s)	59.8 ± 29.1	84.1 ± 25.5	54.1 ± 17.7	96.1 ± 23.5	<0.001

Data are presented as mean ± standard deviation (SD). Comparisons among the four phenotypes were performed using one-way analysis of variance (ANOVA).

BMI, body mass index; AHI, apnea–hypopnea index; SpO₂, oxygen saturation; LAP, longest apnea duration; LHP, longest hypopnea duration.

Significant differences were observed among the four phenotypes in the clustering variables, including demographic characteristics, obesity-related parameters, disease severity, nocturnal oxygenation, and respiratory event duration (all *P* < 0.001). Because these variables were used to construct the clusters, these between-cluster differences should be interpreted as descriptive characteristics of the derived phenotypes rather than independent evidence of cluster validity. Cluster 1 (*n* = 462, 48.2%) represented the mild phenotype. This cluster was characterized by the lowest AHI, the highest lowest oxygen saturation, and the shortest longest apnea duration (LAP). Body mass index (BMI) and neck circumference were also lower than those observed in the remaining phenotypes. Cluster 2 (*n* = 275, 28.7%) represented the obesity-associated severe phenotype. Patients in this cluster exhibited the highest BMI, the largest neck circumference, the highest AHI, and the lowest oxygen saturation among all four phenotypes. Both LAP and the longest hypopnea duration (LHP) were also prolonged. Cluster 3 (*n* = 107, 11.2%) demonstrated an intermediate level of disease severity. Although the AHI was only moderately elevated, LAP was markedly prolonged, whereas LHP remained relatively short compared with the other phenotypes. Cluster 4 (*n* = 115, 12.0%) exhibited the longest respiratory event duration among all phenotypes. Despite an intermediate AHI level, both LAP and LHP were the longest, accompanied by more pronounced nocturnal oxygen desaturation than that observed in the mild phenotype. Overall, respiratory event duration provided additional temporal characterization across phenotypes with different levels of conventional AHI-based severity.

### Pairwise comparisons among the four phenotypes

Post hoc Bonferroni analyses further characterized the differences among the four phenotypes (Table 2). Significant pairwise differences were observed for most demographic and polysomnographic variables. Cluster 2 exhibited significantly higher BMI and larger neck circumference than Clusters 1, 3, and 4 (all adjusted *P* < 0.001). No significant differences in BMI or neck circumference were observed between Clusters 1 and 3 or between Clusters 3 and 4. AHI differed significantly between every pair of phenotypes (all adjusted *P* < 0.01), indicating that the four phenotypes also differed in conventional disease severity. Respiratory event duration showed the greatest discriminatory ability among the four phenotypes. LAP differed significantly between every pair of clusters (all adjusted *P* < 0.001), increasing progressively from Cluster 1 to Cluster 4. In contrast, LHP followed a different pattern, with the longest duration observed in Cluster 4, followed by Cluster 2, whereas no significant difference was found between Clusters 1 and 3 (*P* = 0.275). Lowest oxygen saturation also differed significantly among all phenotype pairs, demonstrating progressively worsening nocturnal hypoxemia from the mild phenotype to the more severe phenotypes.

**Table 2:** Bonferroni-adjusted pairwise comparisons among the four OSA phenotypes.

Variable	C1 vs. C2	C1 vs. C3	C1 vs. C4	C2 vs. C3	C2 vs. C4	C3 vs. C4
Age	<0.001	0.055	<0.001	1.000	<0.001	1.000
BMI	<0.001	1.000	0.236	1.000	<0.001	1.000
Neck circumference	<0.001	1.000	0.872	1.000	<0.001	1.000
AHI	<0.001	<0.001	<0.001	<0.001	<0.001	0.009
Lowest SpO₂	<0.001	<0.001	<0.001	<0.001	0.022	<0.001
LAP	<0.001	<0.001	<0.001	<0.001	<0.001	<0.001
LHP	<0.001	0.275	<0.001	<0.001	<0.001	<0.001
Hypopnea index	<0.001	0.015	<0.001	<0.001	<0.001	<0.001

Pairwise comparisons were calculated from the original dataset using the same four-cluster grouping (QCL_2) and the pooled within-group error term from one-way ANOVA. Two-sided *P* values were multiplied by 6 for the six pairwise comparisons (Bonferroni adjustment), with values capped at 1.000000. The calculations reproduced the previously reported adjusted *P* = 0.054864 for age (C1 vs. C3) and *P* = 0.275072 for LHP (C1 vs. C3).

C1–C4, Cluster 1–Cluster 4; LAP, longest apnea duration; LHP, longest hypopnea duration; SpO₂, oxygen saturation.

Overall, pairwise comparisons showed that the phenotypes differed in respiratory event duration as well as conventional severity measures. These findings support the use of LAP and LHP as complementary descriptive dimensions within the exploratory phenotype classification.

### Visualization of the four phenotypes

Principal component analysis (PCA) provided a two-dimensional visualization of the four phenotypes (Figure 3). Cluster 1 and Cluster 2 were primarily separated along the first principal component, whereas Clusters 3 and 4 showed greater separation along the second principal component. Although partial overlap was observed among the four clusters, the PCA plot visually illustrated the multidimensional structure of the derived phenotypes. The overlap between Clusters 3 and 4 also indicates that PCA should be regarded as a visualization tool rather than an independent validation of clustering robustness.

**Figure 3:**
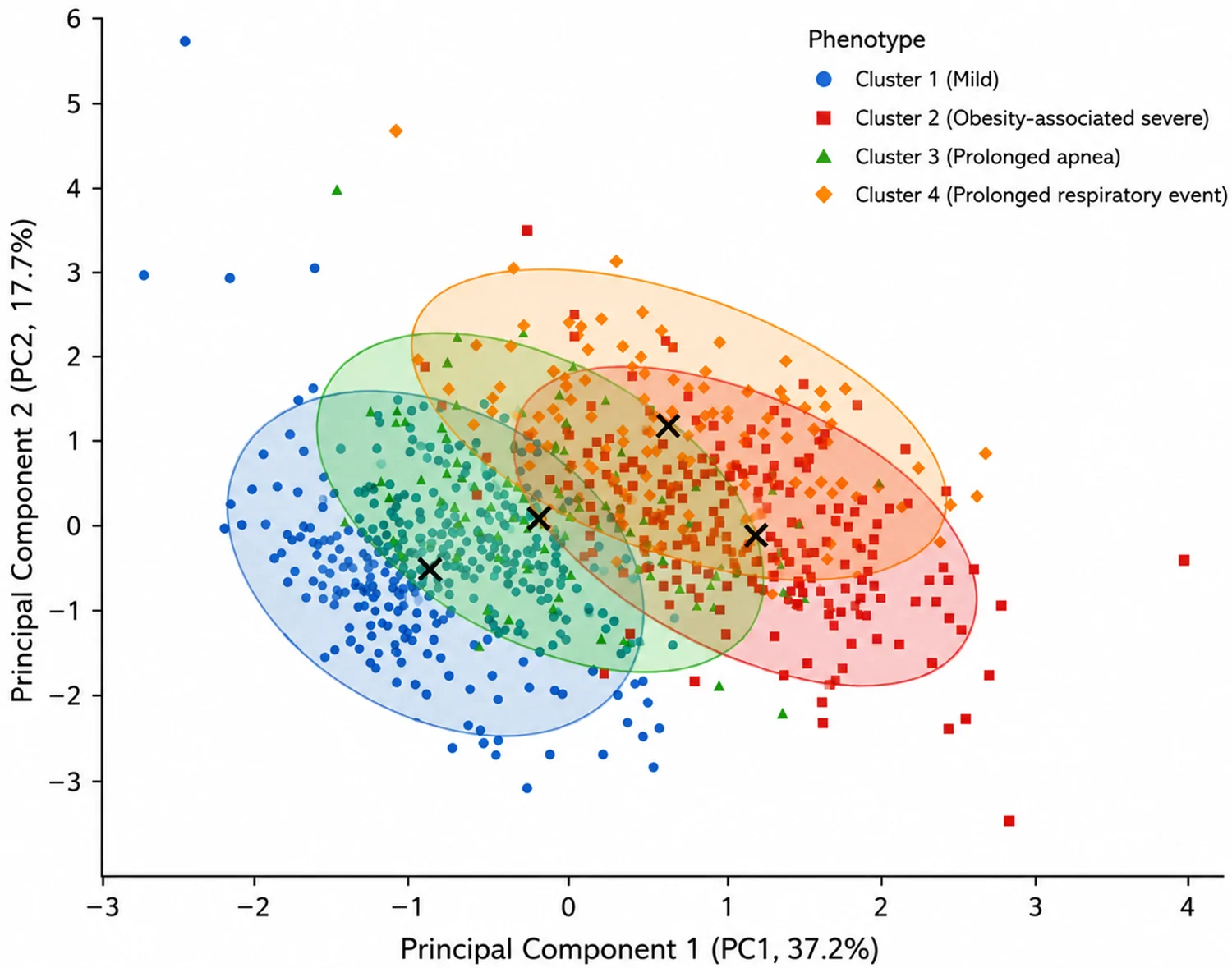
Principal component analysis visualization of the four exploratory OSA phenotypes. PC1 and PC2 explained 37.2% and 17.7% of the total variance, respectively. Each point represents an individual participant, and black crosses indicate cluster centroids. The ellipses illustrate the distribution of observations within each cluster. PCA was used for visualization of the derived clusters and was not considered an independent validation of cluster stability.

## Discussion

### Principal findings

Using hierarchical and K-means cluster analyses, we identified four exploratory OSA phenotypes in a large cohort of adults undergoing diagnostic polysomnography. In addition to conventional measures of disease severity, the clustering framework incorporated respiratory event duration, particularly the longest apnea duration (LAP) and longest hypopnea duration (LHP). The resulting phenotypes differed in obesity-related characteristics, conventional disease severity, nocturnal oxygenation, and respiratory event duration, illustrating the multidimensional structure of OSA.

### Respiratory event duration represents an additional dimension of OSA heterogeneity

Conventional assessment of OSA severity relies predominantly on event frequency, which is quantified by the apnea–hypopnea index (AHI) (9, 10). Although the AHI has long served as the cornerstone of OSA diagnosis and severity classification, it provides little information regarding the temporal characteristics of individual respiratory events (10). Consequently, patients with similar AHI values may experience substantially different cumulative hypoxic burdens and respiratory disturbances during sleep (13, 14).

Our findings support this concept by showing that respiratory event duration, particularly LAP and LHP, distinguished phenotypes with different temporal characteristics that were not captured by AHI alone. Clusters 3 and 4 had intermediate AHI values but markedly different respiratory event durations. These observations suggest that respiratory event duration may represent a complementary physiological dimension of OSA heterogeneity beyond conventional event frequency (15, 23).

Growing evidence suggests that respiratory event duration is an underappreciated physiological characteristic of OSA. Compared with shorter respiratory events, prolonged events may be associated with greater hypoxic burden and more pronounced physiological consequences, even among patients with comparable AHI values (13, 24, 25). Our findings extend these observations by showing that respiratory event duration can distinguish exploratory OSA phenotypes in conjunction with conventional AHI-based severity measures. The present analysis does not establish that LAP or LHP is independent of AHI.

### Distinct clinical characteristics of the four OSA phenotypes

The four phenotypes identified in the present study exhibited distinct clinical and polysomnographic characteristics, indicating that OSA heterogeneity extends beyond conventional severity classification (18, 20–22). The obesity-associated severe phenotype was characterized by the highest BMI, largest neck circumference, and greatest AHI, consistent with the well-established association between obesity and upper airway collapsibility (25, 26).

In contrast, the prolonged apnea phenotype was characterized by markedly prolonged apnea duration despite only moderate AHI values, whereas the prolonged respiratory event phenotype demonstrated the longest apnea and hypopnea durations together with more severe nocturnal oxygen desaturation. These findings indicate that respiratory event duration does not necessarily increase in parallel with AHI and may therefore capture physiological characteristics that are not reflected by event frequency alone (24).

Anthropometric characteristics also differed substantially among the four phenotypes. Recent clinical data further support the multidimensional nature of OSA severity, showing that neck and waist circumference, symptom burden, and cardiometabolic comorbidities may vary across PSG-defined severity categories, whereas BMI alone may have a more limited association with AHI (27). Patients with the obesity-associated severe phenotype had significantly greater BMI and neck circumference than the remaining phenotypes, whereas prolonged respiratory events were also observed in patients without the highest degree of obesity (28). This finding suggests that mechanisms beyond obesity are likely to contribute to respiratory event duration, including upper airway collapsibility, ventilatory control instability, and arousal threshold (29, 30).

Taken together, these observations suggest that the identified phenotypes reflect different combinations of obesity, respiratory event frequency, oxygenation, and respiratory event duration. Such multidimensional classification may provide a broader descriptive characterization of OSA heterogeneity than severity stratification based solely on AHI. However, whether this exploratory classification offers advantages over conventional AHI-based classification requires prospective validation.

### Clinical implications of respiratory event duration-based phenotyping

The identification of distinct respiratory event duration–based phenotypes may have important clinical implications. Current clinical practice relies predominantly on the AHI to assess disease severity and guide treatment decisions (9, 10, 31). However, patients with similar AHI values may exhibit markedly different respiratory event durations, indicating that conventional severity classification may not fully capture the physiological burden of OSA (32).

In the present study, the prolonged apnea phenotype and the prolonged respiratory event phenotype exhibited substantially prolonged apnea duration despite only moderate differences in AHI, suggesting that respiratory event duration provides complementary information beyond event frequency alone. Prolonged respiratory events are likely to increase cumulative hypoxic exposure and delay physiological recovery during sleep, potentially resulting in biological consequences that differ from those associated with comparable AHI values (14, 15).

Because respiratory event duration can be obtained from routine polysomnography without additional examinations, these parameters may be feasible as complementary descriptors in PSG reporting (31, 33). However, the present study did not evaluate treatment response, cardiovascular outcomes, daytime symptoms, or long-term prognosis, and therefore cannot establish that duration-based phenotyping improves risk stratification or individualized management. Future prospective and multicenter studies incorporating hypoxic burden, symptoms, comorbidities, and treatment outcomes are needed to determine its clinical utility (34).

### Strengths and limitations

Several features of this study strengthen the interpretation of our findings. First, we analyzed a relatively large cohort of adults with OSA who underwent standardized overnight diagnostic polysomnography using the same recording system and scoring criteria, thereby minimizing methodological heterogeneity. Second, unlike previous phenotype studies that focused primarily on conventional clinical characteristics or AHI-based severity, we incorporated respiratory event duration, including LAP and LHP, into the clustering model, providing a novel perspective for characterizing OSA heterogeneity. Third, the combined use of hierarchical clustering, K-means clustering, and principal component analysis provided complementary approaches for deriving and visualizing the exploratory phenotypes; PCA was used for visualization rather than independent validation. To our knowledge, this is among the first studies to integrate respiratory event duration into PSG-based phenotype classification.

The findings should also be interpreted in light of several limitations. First, this was a retrospective single-center study, which may limit the generalizability of our findings to other populations. Second, the present clustering analysis does not establish incremental predictive value or independence of LAP or LHP from AHI, and important clinical outcomes, including daytime sleepiness, cardiovascular comorbidities, hypoxic burden, and treatment response, were not evaluated. Third, 40 patients were excluded because of missing clustering variables, with missingness concentrated in LAP and LHP; complete-case analysis may therefore have introduced selection bias. LAP and LHP are maximum-duration measures and may also be influenced by the number of respiratory events, sleep duration, sleep stage, body position, scoring characteristics, and individual unusual events. Finally, the clustering model was internally derived and was not externally validated or subjected to formal resampling-based stability analysis. Future studies should integrate physiological traits, longitudinal follow-up, and clinically relevant outcomes to determine the reproducibility and clinical applicability of these phenotypes.

## Conclusions

In conclusion, hierarchical and K-means clustering identified four exploratory PSG-based OSA phenotypes with distinct combinations of conventional severity measures, oxygenation, anthropometric characteristics, and respiratory event duration. LAP and LHP provided complementary descriptive information about the temporal characteristics of respiratory events but should not be interpreted as replacements for AHI or as independently validated predictors. External validation and prospective studies linking these phenotypes to hypoxic burden, symptoms, treatment response, and clinical outcomes are needed before their clinical utility can be established.

## Data Availability

The raw data supporting the conclusions of this article will be made available by the authors, without undue reservation.
